# Azumamides A-E: Isolation, Synthesis, Biological Activity, and Structure–Activity Relationship

**DOI:** 10.3390/molecules27238438

**Published:** 2022-12-02

**Authors:** Sooheum Jo, Jin-Hee Kim, Jiyeon Lee, Youngjun Park, Jaebong Jang

**Affiliations:** 1 College of Pharmacy, Korea University, Sejong 30019, Republic of Korea; 2 College of Pharmacy, Yonsei Institute of Pharmaceutical Sciences, Yonsei University, Incheon 21983, Republic of Korea; 3 Laboratory of Immune and Inflammatory Disease, Jeju Research Institute of Pharmaceutical Sciences, College of Pharmacy, Jeju National University, Jeju 63243, Republic of Korea; 4 Interdisciplinary Graduate Program in Advanced Convergence Technology and Science, Jeju National University, Jeju 63243, Republic of Korea

**Keywords:** azumamide, naturally occurring cyclic peptide, HDAC inhibitor, β-amino acid, asymmetric synthesis, macrocyclization

## Abstract

Cyclic peptides are one of the important chemical groups in the HDAC inhibitor family. Following the success of romidepsin in the clinic, naturally occurring cyclic peptides with a hydrophilic moiety have been intensively studied to test their function as HDAC inhibitors. Azumamides A-E, isolated from *Mycale izuensis*, are one of the powerful HDAC inhibitor classes. Structurally, azumamides A-E consist of three *D*-α-amino acids and unnatural β-amino acids such as 3-amino-2-methyl-5-nonenedioic acid-9-amide (Amnna) and 3-amino-2-methyl-5-nonenoic-1,9-diacid (Amnda). Moreover, azumamides have a retro-arrangement peptide backbone, unlike other naturally occurring cyclopeptide HDAC inhibitors, owing to the *D*-configuration of all residues. This review summarizes the currently available synthetic methods of azumamides A-E focusing on the synthesis of β-amino acids and macrocyclization. In addition, we overview the structure–activity relationship of azumamides A-E based on reported analogs. Collectively, this review highlights the potentiality of azumamides A-E as an HDAC inhibitor and provides further developmental insight into naturally occurring cyclic peptides in HDAC inhibition.

## 1. Introduction

Unlike prokaryotes, eukaryotic cells have specialized components that confer chromatin condensation for the stability and dynamics of the genome. This process is mainly mediated by histone proteins [1]. The histone family consists of five small proteins that are charged positively [2,3]. H2A, H2B, H3, and H4 are core units where negative-charged deoxyribonucleic acid (DNA) winds around to form the nucleosome. Then, the H1 protein binds the nucleosome to form the chromatosome, consequently building the chromosome through repetitive coiling of the chromatosome [4,5,6]. To acquire genetic information through the action of transcriptional/translational machineries on DNA, compact DNA packaging has to be first untangled. This process is generally governed by the modification of DNA and histone proteins, which is referred to as epigenetics [7].

For DNA epigenetic regulation, DNA is chemically modified by DNA methyltransferases (DNMTs) that catalyze the binding of the methyl group to the DNA base. For example, DNMTs translocate the methyl group of S-adenosyl methionine to cytosine, leading to the production of 5-methylcytosine. This methylated form of cytosine is among the earliest detected types of epigenetically modified DNA and is elucidated to repress gene expression [8,9]. In the context of histone proteins, lysine acetylation at histone tails plays an integral role in modulating chromatin status [10]. Histone acetylation transforms closed chromatin into open form by inducing a conformational change of structure, which transcriptionally promotes gene expression [10]. Given that multiple pathological conditions, such as cancer, autoimmunity, and aging, display aberrant patterns of DNA methylation/histone acetylation, targeting epigenetic regulators has been considered as a promising strategy for desperate patients [11,12].

Of note, histone acetylation is one of the key epigenetic modifications, of which the mechanism involves transferring an acetyl moiety from acetyl-CoA to the lysine ε-amino group on the histone via covalent bond formation. The addition and elimination of the acetyl group in the histone are mediated by histone acetyltransferase (HAT) and histone deacetylase (HDAC), respectively. In this context, HDAC inhibitors have been intensively studied, and several HDAC inhibitors have already been approved by the US FDA, such as vorinostat, belinostat, romidepsin, tucidinostat, and panobinostat [13].

**Figure 1:**
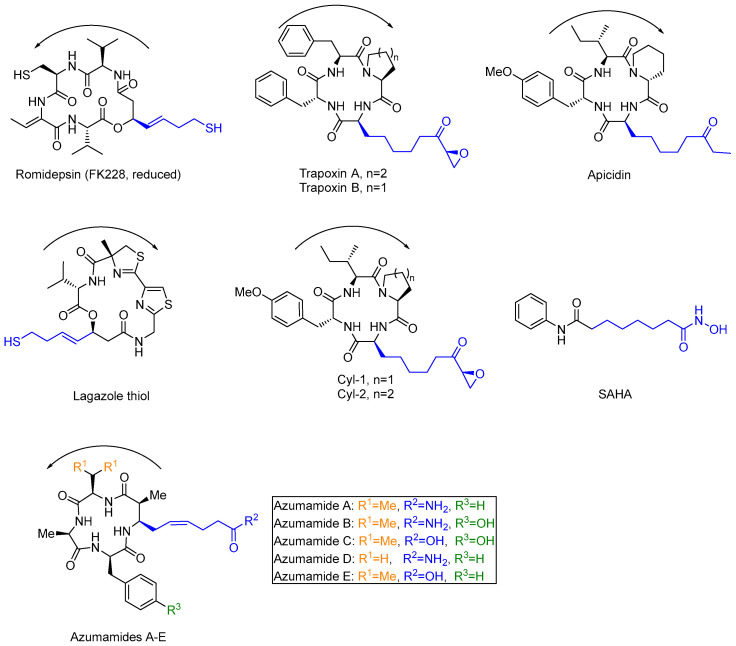
Examples of naturally occurring cyclic peptides characterized as HDAC inhibitors. Direction of N- to C-termini is indicated by arrow. The zinc-binding group is indicated by blue color.

Cyclic peptides are one of the important chemical classes in the drug discovery field, and more than 40 cyclic peptide drugs are currently available in the clinic [14]. Typically, naturally occurring cyclic peptides have attracted attention, which has led to the clinical development of cyclic peptides derived from natural products. One of the examples is romidepsin (FK 228) (Figure 1), a natural cyclic depsipeptide, isolated from the fermentation broth of *Chromobacterium violaceum* [15]. Romidepsin is a type I histone deacetylase (HDAC) inhibitor [16] and has been approved by the FDA for the treatment of patients with cutaneous T-cell lymphoma in 2009. After the success of romidepsin, diverse naturally occurring cyclic peptides were tested in HDAC inhibition. The azumamide family is one of the representative HDAC inhibitors, which are naturally occurring cyclic peptides. All azumamides have been drawing attention in the drug discovery field because they show strong HDAC inhibition and have interesting structural features such as unnatural amino acids with R-configuration and β-amino acid [17].

In this review, we summarize the isolation and structural determination; synthetic methods of β-amino acids, such as 3-amino-2-methyl-5-nonenedioic acid, 9-amide (Amnna), and 3-amino-2-methyl-5-nonenoic-1,9-diacid (Amnda); macrocyclization; and structure–activity relationship analysis of azumamides.

## 2. Isolation and Structural Determination

In the course of exploring antitumor agents in 167 species of Japanese marine invertebrates, azumamides A-E were isolated from Mycale izuensis by Fusetani et al. [17]. Azumamide A was isolated as a colorless solid with an optical activity $({\left[\alpha \right]}_{D}^{23}$ = +33°). The proton signals at δ = 7.63, 7.85, 8.00, and 8.15 ppm in ^1^H-NMR spectroscopy indicated the NH of peptide bonds and δ = 3.60, 4.13, 4.17, and 4.29 ppm showed the signals of the proton at the α-position of amino acids. The structures of four α-amino acids, including alanine (Ala), valine (Val), phenylalanine (Phe), and one β-amino acid, were determined by intensive two-dimensional NMR analysis. The sequence of four amino acids was revealed by analysis of HMBC and ROSEY spectra, resulting in the order of Val-Ala-Phe-β-amino acid. The absolute configuration of three α-amino acids was determined by Marfey’s analysis, in which l-fluoro-2,4-dinitrophenyl-5-l-alanine amide reacts with D- or L-amino acid resulting in the formation of diastereomers [18]. In reverse-phase HPLC separation, D-diastereomer is normally eluted slower than L-diastereomer. The structure of β-amino acid was assigned as 3-amino-2-methyl-5-nonenedioic acid, 9-amide (Amnaa), of which the double bond showed the coupling constant of 11 Hz indicating Z-geometry and placed on C2 and C3 determined by COSY analysis. The terminus of the side chain was an amide group determined by HMBC and HOHAHA spectra. The stereochemistry of the Amnaa was elucidated using the derivative of the methyl ester with (+)-MTPACl after hydrogenation of olefin and hydrolysis. As a result, the 2S,3R-configuration was revealed by comparing the spectrum of this derivative with four stereoisomers of 3-amino-2-methylhexanoic acids. The structure of azumamide B was almost identical to azumamide A except for Tyr instead of Phe in azumamide A, which was simply determined by comparison of 1H-NMR and mass spectrum of azumamides A and B. Azumamide C also has Tyr-like azumamide B, but the terminal group of the side chain was the carboxylic acid assigned by ^1^H-NMR spectrum. Therefore, the β-amino acid of azumamide C was 3-amino-2-methyl-5-nonenoic-1,9-diacid (Amnda), which was also found in azumamide E, having Phe instead of Tyr. Azumamide D has an Amnaa and Phe unit instead of Tyr, and it also has one more Ala instead of Val, determined by ^1^H-NMR and mass spectrum (Figure 1).

## 3. Synthesis of Azumamides A-E

### 3.1. Synthesis of β-Amino Acids Amnna and Amnda

One of the most challenging steps in azumamides synthesis was stereoselective synthesis of β-amino acids Amnna and Amnda. Establishing two chiral centers of β-amino acids was mediated by three different approaches, including stereoselective Brown crotylation [19], Ellman-type Mannich reaction [20,21], or Sharpless asymmetric epoxidation/stereo- and regioselective epoxide opening [10]. For the construction of Z-olefin, Wittig olefination [19,20,21] or partial reduction of the triple bond [22] was utilized. This review summarizes synthetic approaches of these β-amino acids, which were reported by four different research groups.

#### 3.1.1. Synthesis of β-Amino Acid via a Stereoselective Brown’s Crotylboration and Wittig Olefination

**Scheme 1:**
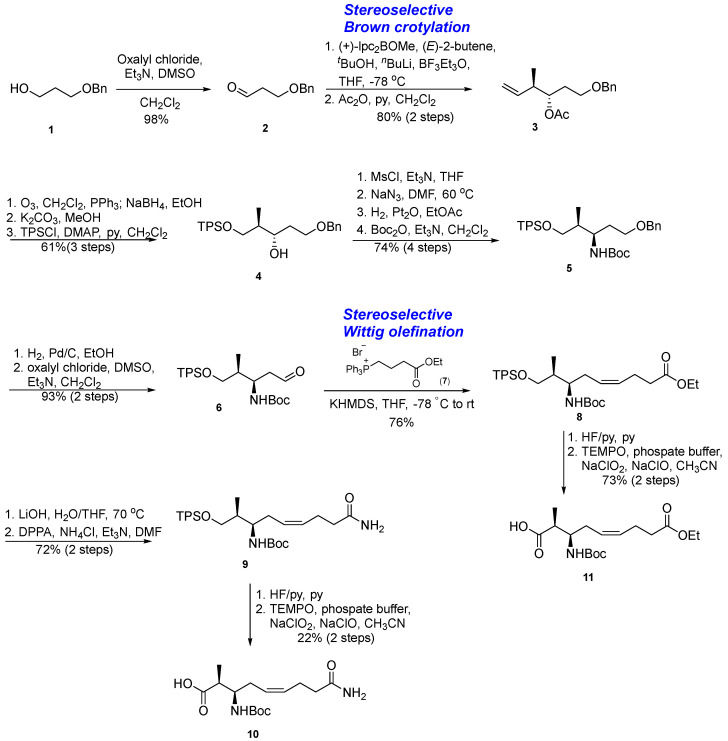
Synthetic approach of β-amino acid developed by Izzo and De Riccardis et al.

Izzo and De Riccardis et al. reported the first total syntheses of azumamides A and E in 2006 [19]. In this synthesis, the β-amino acid Amnaa was synthesized via a diastereo- and enantioselective Brown crotylboration reaction to obtain two key chiral centers and a highly stereoselective Wittig olefination to produce (*Z*)-olefin (Scheme 1). First, the aldehyde 2 was obtained by oxidation of 3-benzyloxypropanol 1 in the presence of oxalyl chloride and dimethyl sulfoxide. The intermediate 3 containing two key stereogenic centers was obtained through a highly stereoselective crotylation by reacting the aldehyde 2 and the chiral reagent (+)-(*E*)-crotyl-Ipc2-borane with >99% d.r. and 98% ee. Three modification steps produced a regioselectively silylated triol 4, and a stereoselective amination by mesylation, substitution, and reduction, followed by Boc-protection, produced an orthogonally protected aminodiol 5. A stereoselective Wittig olefination provided the ester 8 by reacting the ylide prepared from the phosphonium salt 7 and the aldehyde 6 obtained by debenzylation and Swern oxidation of the amino diol 5. Only the *Z*-olefin was detected in ^1^H-NMR spectroscopy in good yield (76%). The ester 8 was transformed to the amide 9 via ester hydrolysis and amide formation using DPPA and ammonium chloride. A drawback of the approach developed by Izzo and De Riccardis et al. was that the final oxidation of the primary alcohol, obtained after desilylation of the intermediate 9, exhibited only 22% yield (2 steps). The overall yield of the last two steps (desilylation and oxidation) was improved by 73% when the reactions proceeded with the terminal ethyl ester functionality instead of the corresponding carboxamide.

#### 3.1.2. Synthesis of β-Amino Acid via Mannich Reaction with Ellman’s *tert*-Butylsulfinyl Auxiliary 

**Scheme 2:**
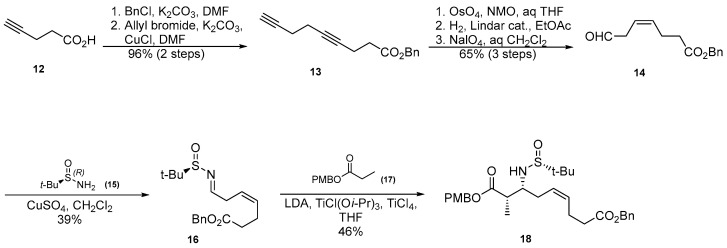
Synthetic approach of β-amino acid developed by Ganesan et al.

Ellman’s *tert*-butylsulfinyl auxiliary has been broadly used in the synthesis of unnatural amino acids [23]. Ganesan et al. applied a stereoselective Mannich reaction using Ellman’s *tert*-butylsulfinyl auxiliary 15 to synthesize the Amnaa, which commenced with the conversion of an acetylene 11 to a β,γ-unsaturated aldehyde 14 via Martin’s four-step sequence [24] including allylation, dihydroxylation, partial hydrogenation, and oxidative cleavage (Scheme 2) [20]. CuSO_4_-mediated dehydration reaction using Ellan’s *tert*-butylsulfinyl auxiliary 15 led to conversion of the aldehyde 14 to a key intermediate, imine 16. Stereoselective Mannich reaction with a propionate ester enolate afforded β-amino acid 18, of which configuration was confirmed using a saturated analog via Mosher’s ester method. This approach proceeded with a high diastereoselective manner, while the yields of the imine formation and Mannich reaction steps were slightly low at 39% and 46%, respectively, due to the instability of the aldehyde 14 and imine 16. Orthogonally tri-protected β-amino acid 18 was synthesized from the commercially available acetylene 12 in seven steps at 11% overall yield.

#### 3.1.3. Synthesis of β-Amino Acid via an Asymmetric Epoxidation, Diastereo- and Regioselective Epoxide Opening, and Partial Reduction 

**Scheme 3:**
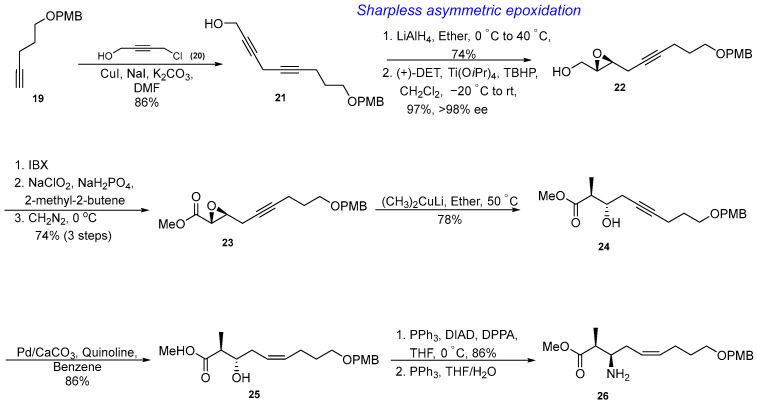
Synthetic approach of β-amino acid developed by Chandrasekhar et al.

An asymmetric epoxidation, diastereo- and regioselective epoxide opening, and a partial reduction of the triple bond were applied to synthesize the Amnaa (Scheme 3) [22]. The synthesis commenced with the PMB protection of propargylic alcohol, followed by coupling with 20 in the presence of CuI, NaI, and K_2_CO_3_. The propargylic alcohol 21 was selectively reduced by LiAlH_4_ to obtain (*E*)-allyl alcohol, which was exposed in the Sharpless asymmetric epoxidation resulting in the formation of 22 at 97% yield and >98% ee. After obtaining the epoxy ester 23 via the oxidation of the primary alcohol in two steps and esterification with diazomethane, a regio- and stereoselective epoxide opening was performed with the methyl cuprate generated following the literature procedure [25]. As a result, two chiral centers were successfully generated with excellent stereoselectivity. Then, the partial hydrogenation of 24 using Lindlar’s catalyst established the key functional group (*Z*)-olefin. Mitsunobu reaction with DPPA, followed by the reduction of the azide group, generated β-amino acid 26 with inversion of the configuration. The PMB-protected terminal alcohol was converted to a carboxylic acid at the late stage after the construction of the cyclic tetrapeptide via PMB deprotection and oxidation in the presence of BAIB and TEMPO.

#### 3.1.4. Synthesis of β-Amino Acid via Ellman-Type Mannich Reaction and Wittig Olefination

**Scheme 4:**
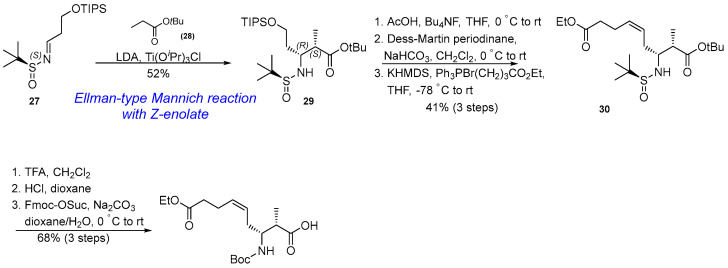
Synthetic approach of β-amino acid developed by Olsen et al.

The Ellman-type Mannich reaction was utilized again by Olsen et al. to establish two key chiral centers of β-amino acid, Amnaa [21]. They performed this reaction at the early stage with a simple substrate 27, and the substrates and reaction conditions were optimized to obtain proper stereochemistry and improved diastereo- and enantioselectivities (Scheme 4). Unlike Ganesan’s approach [20], in which the Ellman auxiliary with *R*-configuration led to (2*S*, 3*R*)-β-amino acid, this simple substrate led to an undesired stereochemistry outcome with (2*S*, 3*S*)-configuration, determined by the X-ray crystal structure, which was presumably due to the formation of an unexpected transition state. After intensive optimization, the desired configuration (2*S*, 3*R*) was obtained when the sulfinylimine 27 with an opposite configuration (*S*) and *Z*-ester enolate, synthesized using Ireland’s condition, were used. Like Izzo’s approach, Wittig olefination with a proper ylide was applied to obtain a protected Amnda 30. Finally, manipulation of the protecting groups and removal of the chiral auxiliary led to a Fmoc-protected Amnda 31, at 15% overall yield starting from the imine 27.

### 3.2. Macrocyclization of Azumamides A-E

**Figure 2:**
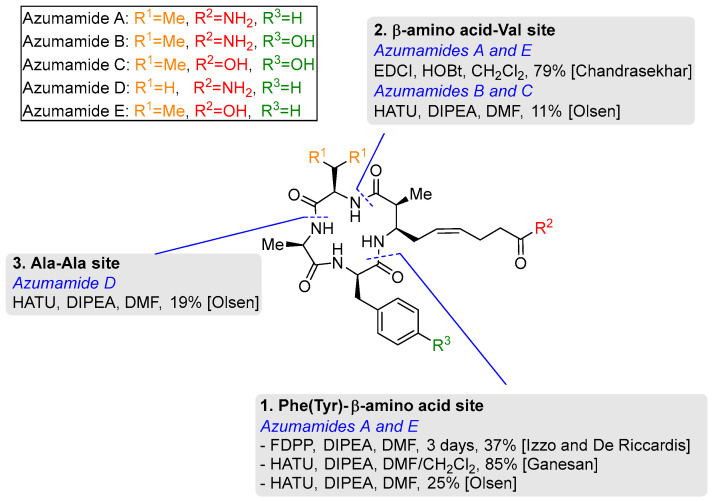
Macrocyclization of azumamides A-E.

After properly protecting Amnaa or Amnda in hands, the rest of the synthesis could be completed via iterative amide coupling reactions and terminal functional group modification. However, one of the difficult steps in the total synthesis of cyclic peptides is the macrocyclization step because of an entropically disfavorable reaction and the requirement of a defined pre-cyclization conformation before cyclization. Generally, several cyclization sites are tested when the classical amidation or esterification is used. In the case of azumamide synthesis, the macrocyclization was successfully performed at three different sites at 11–85% yields (Figure 2).

#### 3.2.1. Phe (Tyr)–β-Amino Acid Site

First, azumamides A and E were synthesized through macrocyclization mostly at the Phe-Amnaa/Amnda site. A tetrapeptide containing Amnaa was utilized in the first attempt to synthesize azumamide A via macrocyclization. However, none of the coupling reagents, such as DPPA, FDPP, and EDC/HOBt, afforded the desired azumamide A [19]. Therefore, azumamide E was first synthesized using an Amnda ethyl ester, lacking the terminal carboxamide prior to converting the terminal carboxylic acid to the carboxamide at the late stage for the synthesis of azumamide A. A linear tetrapeptide containing the Amnda ethyl ester was successfully transformed to the azumamide E ethyl ester by FDPP-mediated macrocyclization at 37% yield [19]. Finally, the first target molecule, azumamide E, was obtained via hydrolysis of the resulting ethyl ester. Subsequently, azumamide A was created via DPPA-induced amidation of azumamide E in the presence of triethylamine and ammonium chloride at 54% yield [19]. After this work was published, terminal amide-free β-amino acid derivatives were utilized in the macrocyclization of the azumamide series. Olsen et al. also cyclized a tetrapeptide with Amnda ethyl ester, but used HATU as a coupling reagent instead of FDPP, leading to a low yield of 25% [21]. Genesan et al. applied the same coupling reagent and base (HATU/DIPEA) as Olsen et al. used, but they utilized a tetrapeptide including the Amnda 2-trichloroethyl ester at the macrocyclization step resulting in the formation of the target cyclic peptide at 85% yield [20].

#### 3.2.2. β-Amino Acid-Val Site

As aforementioned, Chandrasekhar et al. applied an Amnda analog containing a PMB-protected terminal alcohol to synthesize a cyclic intermediate, which was obtained by macrocyclization of a linear tetrapeptide at the Amnda-Val site using EDCI and HOBt as coupling reagents at 79% yield [22]. Olsen et al. also used this site to synthesize azumamide C obtained at 11% yield by treatment of HATU and DIPEA in DMF [21]. Subsequently, azumamide B was synthesized by transforming the terminal ethyl ester to a carboxamide via hydrolysis of ethyl ester and coupling with ammonia under DIC conditions [21].

#### 3.2.3. Ala-Ala Site

Olsen et al. synthesized all the azumamide family, including azumamide D, which contains additional D-Ala instead of D-Val compared to the other azumamides [21]. To avoid a sterically hindered cyclization site, a linear tetrapeptide with two alanine terminal residues was applied to macrocyclization using a HATU coupling reagent. Although azumamide D has Amnaa, the Amnda ethyl ester was kept in use at the macrocyclization step, and the terminal carboxamide was installed in the late stage like the synthesis of azumamide A. Like the macrocyclization of other azumamides, performed by Olsen et al., the isolation yield was relatively low at 19% although the linear tetrapeptide was fully consumed, and a small amount of the corresponding dimer was formed. There were difficulties in the purification step by the preparative reverse-phase HPLC to obtain the pure cyclized products.

## 4. Biological Activity and Structure–Activity Relationship

### 4.1. Biological Role of Histone Deacetylase

An organism sustains its life via the harmony of multiple biological events arising from the complicated network of chromosomal read-out. DNA winds around histone proteins for compact packaging, which leads to the formation of the nucleosome, a basic unit of chromatin [26]. This packaging forms an extremely condensed structure so that the transcriptional enzyme complex is unable to approach the DNA sequence [27]. Thus, a series of structural modifications that loosen the nucleosome are prerequisite for deciphering the genetic code, which is generally found in histone proteins [28]. Histone acetylation at the lysine residue is one of the most powerful modifications by which DNA becomes permissive to transcription machineries [10]. There are two key enzymes modulating the acetylation of histone proteins: HAT and HDAC. HAT introduces acetyl groups at the ε-amino group of lysine in H3 and H4 histone proteins, while this state is reversed by HDAC that catalyzes the removal of acetyl groups from the N-terminus of lysine in histone proteins, which is correlated with the activation and repression of gene expression, respectively [29].

Of note, a highly conserved HDAC is composed of a group of 18 genes in humans, which are categorized into 4 subtypes: Classes I, II, III, and IV [30]. Classes I, II, and IV are Zn^2+^-containing metalloenzymes, which are also referred to as classical HDAC. Class III enzymes are called sirtuins, and their enzymatic activity is dependent on nicotinamide adenine dinucleotide (NAD^+^) [31]. Commonly, HDAC inhibitors currently being tested in the clinic are targeting classical HDAC. Although HDAC has been initially reported as a deacetylating enzyme of histone proteins, accumulating evidence reveals that HDAC plays an integral role in eliminating acetyl groups from non-histone proteins as post-translational modifications that affect the stability, interaction, and localization of proteins [29]. The significance of HDAC has been well implicated in cancer [32]. For example, promyelocytic leukemia (PML) and retinoic acid receptor α (RARα) proteins are fused to mediate the oncogenesis of acute myeloid leukemia by recruiting HDAC to the target genes of retinoic acid, which are silenced by deacetylation [33]. Moreover, PML- RARα fusion protein mediates HDAC recruitment to tumor suppressor p53 protein [34]. Deacetylated p53 is degraded by the MDM2-dependent proteasome pathway, indicating HDAC-driven p53 inactivation as one of the oncogenic mechanisms. These observations, along with additional studies supporting the tumor-promoting roles of HDAC, shed light on the development of HDAC inhibitors to treat patients with multiple types of cancer [35].

### 4.2. HDAC Inhibition of Azumamides and Structure–Activity Relationship

**Figure 3:**
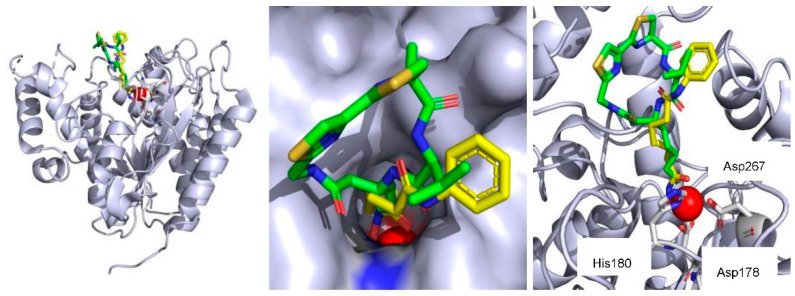
Binding modes of largazole (green) and SAHA (yellow) obtained from the co-crystal structure of HDAC8 complexed with largazole (PDB: 4RN0) or SAHA (PDB: 1T69); Zinc ion (Zn^2+^) presents as a red ball shape. This figure was generated using Pymol.

The structural features of azumamides A-E resemble other naturally occurring HDAC inhibitors such as romidepsin (FK228) [15], trapoxin A/B [36], apicidin [37], largazole [38], and Cyl-1/2 [39,40] (Figure 1). All these cyclic peptides contain a long lipid chain capping with a hydrophilic functional group such as thiol (romidepsin, largazole), epoxy ketone (trapoxin A/B, Cyl-1/2), thioester (largazole), ketone (apicidin), and carboxamide/carboxylic acid (azumamides), which act as a zinc-binding motif, a crucial pharmacophore of HDAC inhibitors. The lipid chain of largazole inserts into a narrow pocket of HDAC8, and the thiol group coordinates with zinc ion with His180, Asp178, and Asp267 at the crystal structure of HDAC8 complexed with largazole thiol [38] (PDB:4RN0) (Figure 3). This lipid chain well overlaps with the lipid chain of SAHA [41] (PDB:1T69), a synthetic HDAC inhibitor, and the thiol is placed at the same position as the hydroxamic acid of SAHA. The macrocycle of largazole is placed at the lip of this narrow pocket and makes contact with the protein surface. Compared to the phenyl ring of SAHA, this macrocycle binds to a wide range of protein surfaces. All naturally occurring HDAC inhibitors including azumamides can have a similar binding mode to that of largazole.

Several studies have reported the HDAC inhibitory activities of azumamides and their analogs [17,20,21,22,42,43,44], which are demonstrated in Table 1. Additionally, this review briefly summarized the understanding of the structure–activity relationship (SAR) to reconcile the results from different groups. The HDAC inhibitory activities of azumamides A-E were first evaluated using enzymes extracted from K562 cells by Fusetani et al., with IC_50_ values of 0.045 μM, 0.11 μM, 0.11 μM, 1.3 μM, and 0.033 μM, respectively [17] (Table 1). HDAC inhibition is highly dependent on the zinc-binding group (ZBG), which also influences isoform selectivity, off-target effects, toxicity, and pharmacokinetic properties [45]. Hydroxamic acid has been broadly used as a ZBG, and HDAC inhibitors containing hydroxamic acid are generally very potent. The natural azumamides showed similar HDAC inhibition regardless of the ZBGs (azumamide A vs. azumamide E; azumamide B vs. azumamide C), but valine analogs (azumamide A-C and E) displayed more than 10-fold stronger HDAC inhibition than the alanine analog (azumamide D). Interestingly, synthetic azumamides showed a different trend in HDAC inhibition. Olsen et al. synthesized and tested all azumamides using a full panel of recombinant human HDAC enzymes [21]. The carboxylic acid analogs azumamides C and E showed strong HDAC inhibitory activities against HDACs 1, 2, 3, 10, and 11, and azumamide C, containing a Tyr residue instead of a Phe residue, was 2-fold more potent than azumamide E, which was the most potent analog among the natural azumamides. The carboxamide analogs azumamides A, B, and D were strikingly inactive against all HDAC enzymes, indicating that carboxylic acid is a better ZBG than carboxamide. A hydroxamic acid analog of azumamide E (Entry 6) synthesized by Ganesan et al. showed improved inhibitory activity against total HDACs extracted from HeLa cells with an IC_50_ value of 0.007 μM, which was 15-fold more potent than synthetic azumamide E (IC_50_ = 0.11 μM) [20]. In the absence of ZBG, the HDAC inhibitory activity was dramatically reduced as expected (Entry 7). These results support that the ZBG is a crucial pharmacophore of the azumamides. The effects of Amnda’s stereochemistry on HDAC inhibition were also evaluated using three azumamide E analogs possessing β2-epi-Amnda, β3-epi-Amnda, or enantiomer of Amnda, respectively (Entries 8–10) [21]. These subtle changes caused a complete loss of potencies. Interestingly, the enantiomer of azumamide E was also almost inactive with an IC_50_ value of 26 μM, compared with the natural azumamide E (IC_50_ = 0.134 μM) tested in the same assays (Entry 11) [42]. Superimposition of natural azumamide E and its enantiomer in the zinc-binding site showed the flipped binding mode of the macrocycles which presumably exerted unfavorable binding interactions [42]. Insertion of an additional methyl group at the β2-position of azumamide E caused a loss of potency at 10 μM concentration (Entry 12), while the elimination of the methyl group at the same position still showed weak HDAC inhibitions with IC_50_ values of 0.6–1.5 μM against HDACs 1–3 and 10–11 (Entry 13) [43]. The saturated Amnda analog was introduced in this desmethyl azumamide E, which was equipotent with the unsaturated one, indicating that the saturation level of the lipid chain can be non-critical in HDAC inhibition (Entry 14). However, the effect of *Z*-olefin on HDAC inhibition should be re-evaluated with a more potent azumamide analog instead of a less active desmethyl compound. Additionally, the introduction of a sugar-mimetic β-amino acid in azumamide E instead of D-Ala caused a dramatic reduction of HDAC inhibition (Entry 15) [22].

## 5. Conclusions

Azumamides A-E from *Mycale izuensis* were initially reported by Nakao and characterized as potent HDAC inhibitors. Subsequently, several chemist groups have reported synthetic methods of azumamides A-E with the asymmetric synthesis of β-amino acids, Amnna and Amnda, and macrocyclization at three different positions. Interestingly, the carboxamide moiety in azumamides A, B, and D was inserted at the late synthetic stage since the formation of a linear peptide with Amnna was inefficient in the macrocyclization step. SAR study was also performed by several groups. In this review, we attempted to summarize the SAR of azumamides A-E and their analogs, although their functions were evaluated in different methods. Mostly, several azumamide analogs with a variation at β-amino acid residues were introduced. Given their enhanced physicochemical and pharmacokinetic properties, cyclic peptides have been considered as better drug candidates than linear peptides. Parallel with romidepsin, azumamides have a retro-arrangement, unlike other naturally occurring cyclic peptide HDAC inhibitors. However, azumamides were less potent than romidepsin against HDACs, presumably due to the inefficient ZBG. Taken together, chemical modification, including the installment of alkyl groups at the amide backbone or switch of α-amino acid to β-amino acid, is required to further improve the ZBG and pharmacokinetic properties, which consequently potentiates the function of azumamides. This will pave the way for the successful development of azumamide analogs that can be potent drug candidates for HDAC inhibition in the clinic.

## Figures and Tables

**Table 1:** HDAC inhibitory activities of azumamides and their analogs ^a^.

Entry	HDAC Inhibitors	Structure	HDAC Inhibition
Entry 1	Azumamide A	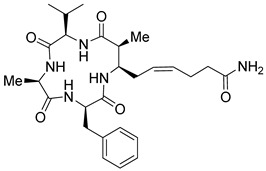	Natural-HDACs ^b^: 0.045 μM [17]
			Synthetic HDAC1 ^c^: >5 μM [21] HDAC2: >5 μM [21] HDAC3: 3.2 μM [21] HDACs: 5.8 μM [20]
Entry 2	Azumamide B	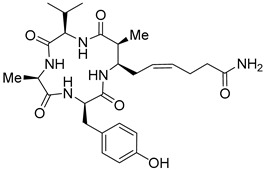	Natural-HDACs: 0.11 μM [17]
			Synthetic HDAC1: 5.0 μM [21] HDAC2: 3.0 μM [21] HDAC3: 3.0 μM [21]
Entry 3	Azumamide C	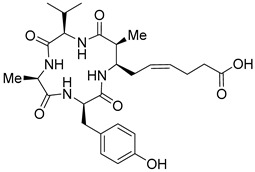	Natural-HDACs: 0.11 μM [17]
			Synthetic HDAC1: 0.032 μM [21] HDAC2: 0.040 μM [21] HDAC3: 0.014 μM [21] HDAC10: 0.010 μM [21] HDAC11: 0.035 μM [21]
Entry 4	Azumamide D	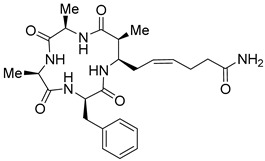	Natural-HDACs: 1.3 μM [17]
			Synthetic HDAC1: >5 μM [21] HDAC2: >5 μM [21] HDAC3: 3.7 μM [21]
Entry 5	(+)-(2*S*,3*R*)-azumamide E [Natural]	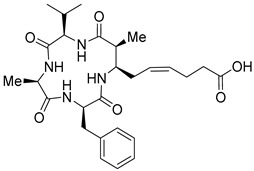	Natural-HDACs: 0.033 μM [17]
			Synthetic HDAC1: 0.067 μM [21] HDAC2: 0.050 μM [21] HDAC3: 0.025 μM [21] HDAC10: 0.020 μM [21] HDAC11: 0.060 μM [21] HDACs: 0.11 μM [20]
Entry 6	Azumamide E hydroxamic acid	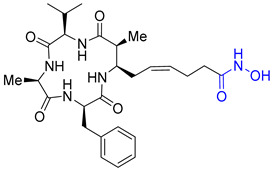	HDACs: 0.007 μM [20]
Entry 7	Carboxylic acid- truncated azumamide C	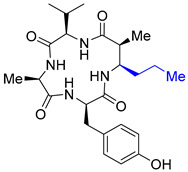	HDAC1: 2.4 μM [44] HDAC2: 1.4 μM [44] HDAC3: 3.0 μM [44]
Entry 8	β^2^-epi-azumamide E	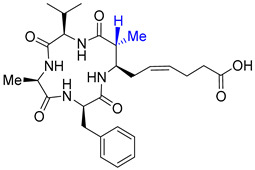	HDAC1: N/A [21] HDAC2: N/A [21] HDAC3: N/A [21]
Entry 9	β^3^-epi-azumamide E	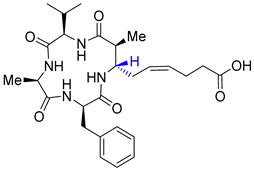	HDAC1: N/A [21] HDAC2: N/A [21] HDAC3: N/A [21]
Entry 10	(2*R*,3*S*)-azumamide E	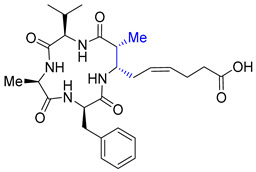	HDACs: N/A at 50 μM [42]
Entry 11	(-)-azumamide E	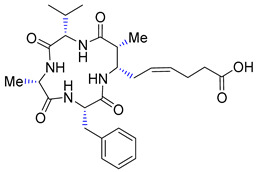	HDACs: 26.0 μM [42]
Entry 12	Dimethyl azumamide E	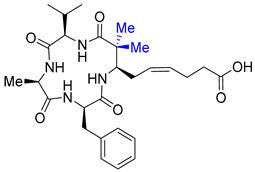	HDAC1: 2–20%inhibition (10 μM) [43] HDAC2: <5%inhibition (10 μM) [43] HDAC3: <5%inhibition (10 μM) [43]
Entry 13	Desmethyl azumamide E	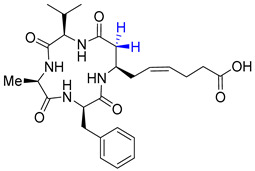	HDAC1: 0.6 μM [43] HDAC2: 0.9 μM [43] HDAC3: 0.8 μM [43] HDAC10: 0.7 μM [43] HDAC11: 1.5 μM [43]
Entry 14	Saturated desmethyl azumamide E	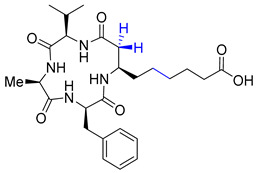	HDAC1: 0.9 μM [43] HDAC2: 0.8 μM [43] HDAC3: 0.7 μM [43] HDAC10: 1.0 μM [43] HDAC11: 2.0 μM [43]
Entry 15	Azumamide E-SAA (sugar amino acid) analogue	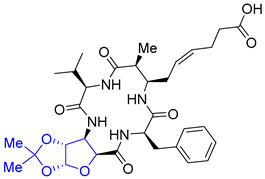	HDACs: 48% inhibition (10 μM); 96% inhibition (20 μM) [22]

^a^ All biological activities of azumamides and their analogs were evidenced by 7 different papers [17,20,21,22,42,43,44]; ^b^ IC_50_ values obtained against total HDACs were evaluated using HeLa cell extracts; ^c^ IC_50_ values obtained against each HDAC isoform were evaluated via biochemical assays using purified HDAC isoforms.

## Data Availability

No new data were created or analyzed in this study. Data sharing is not applicable to this article.
